# Enhanced interstitial fluid drainage in the hippocampus of spontaneously hypertensive rats

**DOI:** 10.1038/s41598-017-00861-x

**Published:** 2017-04-07

**Authors:** Beatrice Bedussi, Daphne M. P. Naessens, Judith de Vos, Rik Olde Engberink, Micha M. M. Wilhelmus, Edo Richard, Malyssa ten Hove, Ed vanBavel, Erik N. T. P. Bakker

**Affiliations:** 1 Department of Biomedical Engineering and Physics, Academic Medical Center, Amsterdam, The Netherlands; 2 Division of Nephrology, Academic Medical Center, University of Amsterdam, Amsterdam, The Netherlands; 3 Department of Anatomy and Neurosciences, Neuroscience Campus Amsterdam, VU medical center, Amsterdam, The Netherlands; 4 Department of Neurology, Academic Medical Centre, Amsterdam, Netherlands; 5 Department of Neurology, Radboud University Medical Centre, Nijmegen, Netherlands

## Abstract

Hypertension is associated with cognitive decline and various forms of dementia, including Alzheimer’s disease. In animal models of hypertension, many of Alzheimer’s disease characteristics are recapitulated, including brain atrophy, cognitive decline, amyloid β accumulation and blood brain barrier dysfunction. Removal of amyloid β and other waste products depends in part on clearance via the brain interstitial fluid (ISF). Here we studied the impact of hypertension on ISF drainage, using spontaneously hypertensive rats (SHR) and normotensive Wistar Kyoto rats (WKY). At 8 months, high (500 kD) and low (3 kD) fluorescent molecular weight tracers released passively into the hippocampus showed a drastically enhanced spreading in SHR. Tracer spreading was inhomogeneous, with accumulation at ISF-CSF borders, around arteries, and towards the stratum lacunosum moleculare. These locations stained positively for the astrocyte marker GFAP, and aquaporin 4. Despite enhanced dispersion, clearance of tracers was not affected in SHR. In conclusion, these data indicate enhanced bulk flow of ISF in the hippocampus of hypertensive rats. ISF drains along astrocytes towards the cerebrospinal fluid compartment, which leads to sieving of high molecular weight solutes. Sieving may lead to a local increase in the concentration of waste products and potentially promotes the aggregation of amyloid β.

## Introduction

Microvascular dysfunction, including impaired neurovascular coupling and blood brain barrier (BBB) disruption, occurs in both vascular dementia and Alzheimer’s Disease^1^. These changes may interfere with the clearance of potentially toxic waste products such as amyloid β, which are released in the brain interstitial fluid (ISF) and accumulate in the parenchyma and vessel walls^1^. Hypertension may aggravate neurodegenerative diseases^2^. Indeed, mid-life hypertension is associated with an increased risk of dementia^3^, while antihypertensive drugs have been found to reduce the risk to develop AD^4, 5^. Induction of high blood pressure by transverse aortic coarctation promotes amyloid β accumulation in the brain of mice^6^. Similarly, work in mouse models of AD showed that hypertension exacerbates AD-like pathology^7–11^. Spontaneously hypertensive rats (SHR) develop white matter damage, cognitive decline (novel object recognition) and BBB disruption, among other pathological changes^12^. Stroke-prone SHR show an age-dependent deposition of amyloid β^13^. Thus, there is a strong link between hypertension and dementia in both human and animal studies. However, the underlying mechanisms for this relation are not well understood. As the clearance of waste products, including amyloid β, from the brain parenchyma depends on transport via the interstitial fluid and subsequent removal via the BBB and paravascular pathways^14–17^, we hypothesized that hypertension may alter fluid dynamics in the brain interstitium. This, in turn, could interfere with the clearance of solutes from the brain. Thus, in the present study we set out to determine the effect of hypertension on distribution and clearance of solutes via the interstitial fluid. For this purpose, we studied the fate of fluorescent tracers released into the ISF of the hippocampus of normotensive and hypertensive rats.

## Results

### Hypertension is associated with a different ionic composition of the brain

Spontaneously hypertensive rats were lighter than normotensive WKY rats (Table 1). Also brain weight was lower in SHR as compared to WKY. As expected, both systolic and diastolic blood pressure were higher in SHR, while also heart rate was significantly increased in SHR as compared to WKY. SHR brains showed a tendency for increased water content, as based on the wet and dry brain weights (Table 1). As edema and altered fluid homeostasis may be more sensitively detected by altered ion concentrations, we measured the concentration of both sodium and potassium. This revealed a significant decrease in potassium content in the SHR brain. As the sodium content showed a small, non-significant increase, a highly significant increase in Na^+^/K^+^ ratio was present in SHR brain.

**Table 1:** Animal characteristics and brain composition.

	WKY	SHR	t-test
**Weight**			
Body (gr)	(n = 20) 404 ± 5	(n = 19) 372 ± 5	p < 0.001
Brain (gr)	(n = 20) 2.26 ± 0.01	(n = 19) 2.12 ± 0.03	p < 0.001
**Blood Pressure**			
Systolic	(n = 20) 163 ± 4	(n = 19) 189 ± 4	p < 0.001
Diastolic	(n = 20) 118 ± 5	(n = 19) 146 ± 4	p < 0.001
BPM	(n = 20) 378 ± 8	(n = 19) 460 ± 5	p < 0.001
**Water content**			
%	(n = 6) 77.155 ± 0.021	(n = 6) 77.612 ± 0.1	0.052
**Ions**			
Na^+^ (mmol/ml Water)	(n = 6) 0.060 ± 0.001	(n = 6) 0.062 ± 0.001	0.117
K^+^ (mmol/ml Water)	(n = 6) 0.111 ± 0.002	(n = 6) 0.106 ± 0.001	0.004
Na^+^ to K^+^ ratio	(n = 6) 0.539 ± 0.012	(n = 6) 0.592 ± 0.011	0.008

SHR body weight and brain weight were lower as compared to WKY. Systolic and diastolic blood pressure, and heart rate were elevated in SHR. Water content in SHR brain tended to be higher as compared to WKY. Whole brain sodium concentration was not different, but the potassium concentration was significantly lower in SHR brain as compared to WKY. Data are mean ± SEM.

### Infusion of tracers into the hippocampus

**Figure 1:**
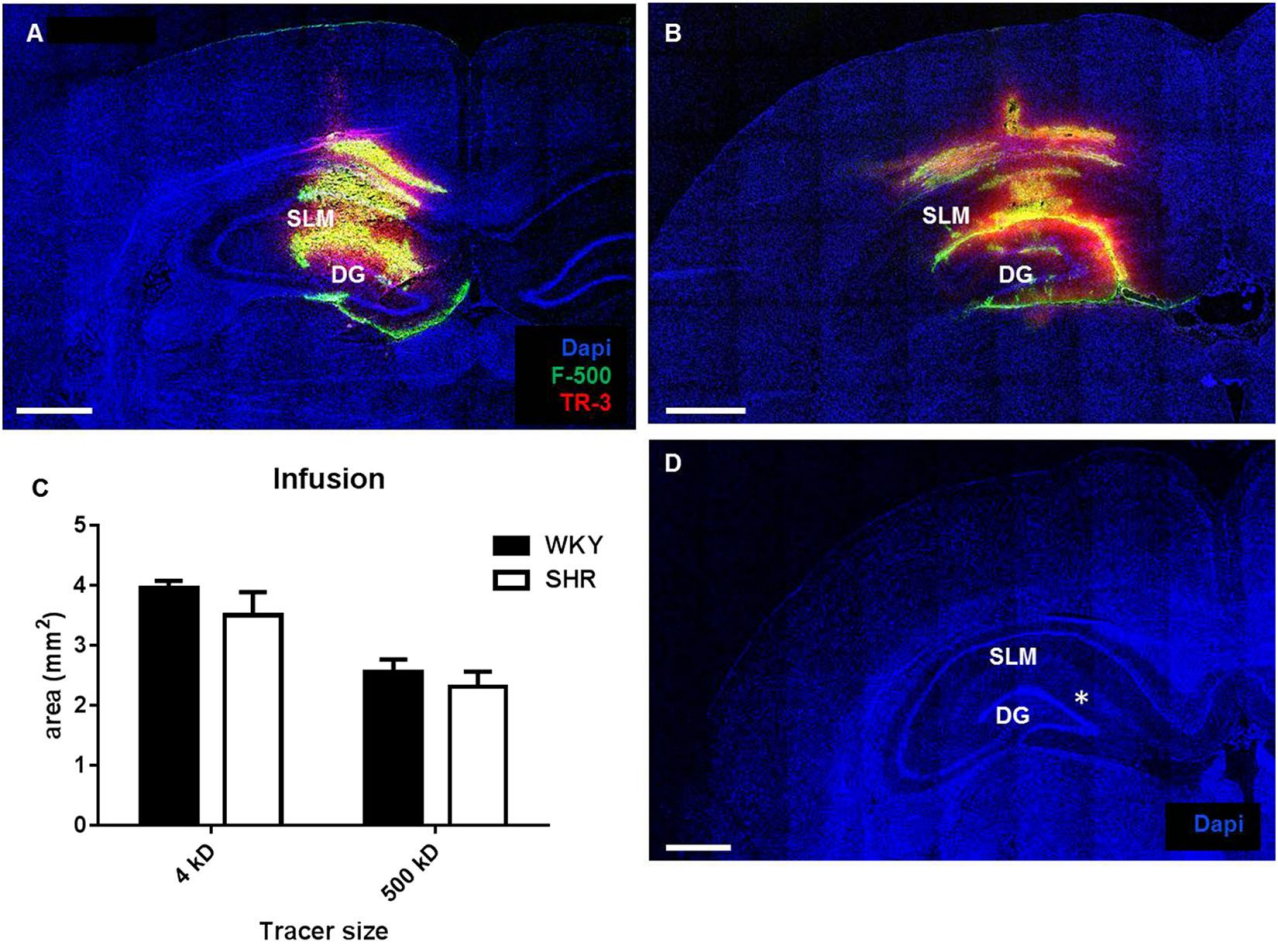
Distribution of fluorescent tracers after infusion into the hippocampus. Panel A and B show coronal slices of WKY (left) and SHR (right) hippocampus at the infusion level. We did not observe a difference in the distribution area of either the low or high molecular weight tracer between WKY (N = 6) and SHR (n = 7). Mean data ± SEM are shown in panel C. In panel D the expected infusion site (*) is shown, based on the stereotactic coordinates for the rat brain. Scale bar 1 mm.

To study the impact of hypertension on the spreading and removal of solutes from the brain extracellular space, we infused a small quantity of fluorescently labelled dextrans into the hippocampus. We infused 2 µl of a mixture of a high and low molecular weight dextran. The combination of a large and a small dextran was used as an indication for diffusion or bulk flow. A large difference in distribution would suggest diffusion, whereas a comparable distribution would suggest bulk flow. For neither tracer did we observe any difference between SHR and WKY (Fig. 1). After using this approach however, we speculated that tracer spreading may have been affected by the infusion itself. We therefore decided to investigate the fate of tracers in the absence of pumping any volume of fluid into the tissue.

### Passive release of tracer in the hippocampus

**Figure 2:**
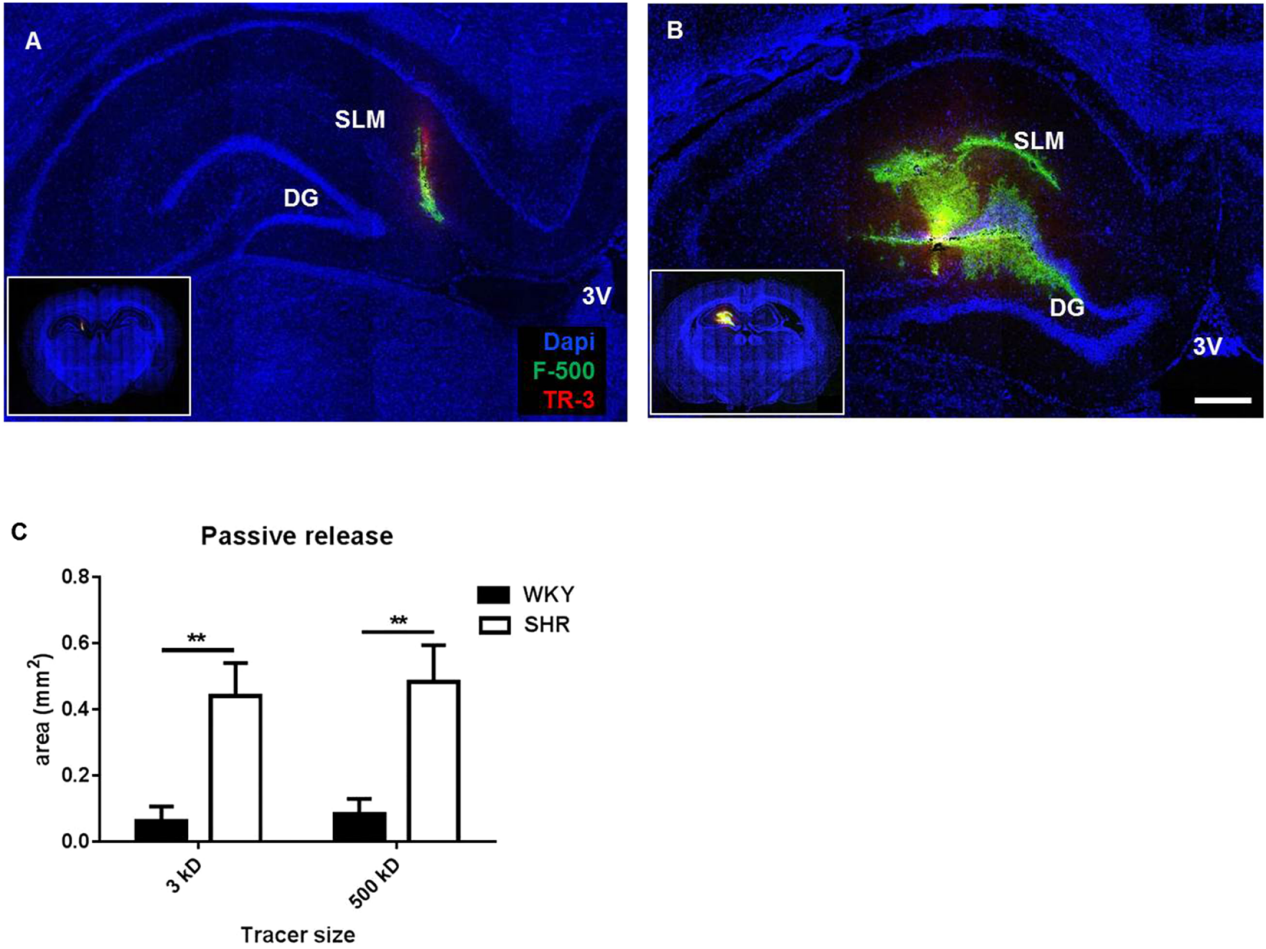
Distribution of tracers after passive release into the hippocampus. Panel A and B show representative coronal sections of WKY (left) and SHR (right) hippocampus at the level of needle insertion. Inserts show the overview of the whole sections. Panel C shows the quantification of the distribution area. Data are mean ± SEM. **Indicates P ≤ 0.001. WKY = 8 SHR = 6. Scale bar 1 mm.

In the second set of experiments we changed our approach and studied tracer spreading after passive release from the needle tip. After stereotactic placement of the needle in the hippocampus, both tracers were allowed to enter the brain from the needle tip by diffusion only, followed by further spreading through the interstitium. Under these conditions, we observed a significant difference in the distribution area between SHR and WKY rats. Thus, both tracers distributed over a 5–6 fold larger area in SHR as compared to WKY (Fig. 2). Remarkably, the distribution area of the small and large tracer was comparable in size for both SHR and WKY. This finding suggests that after release from the needle tip, tracers spread by bulk flow of ISF rather than by diffusion.

### Clearance of tracers from the hippocampus

**Figure 3:**
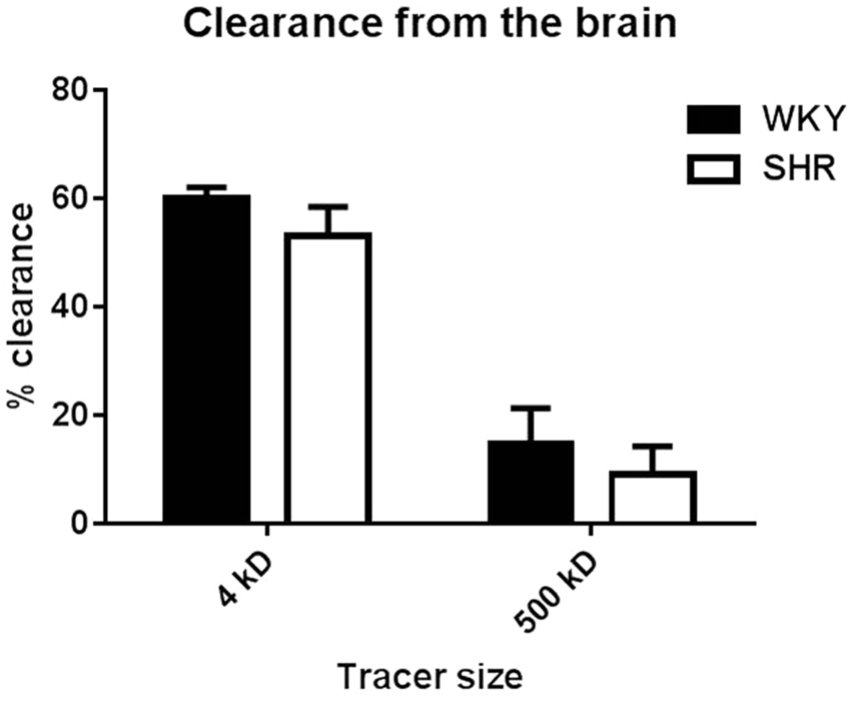
Clearance of tracers from the hippocampus. Clearance was calculated as the amount of tracer removed from the brain as percentage of the initially injected quantity. There was no difference in clearance for both the low and high molecular weight tracer in WKY as compared to SHR. Clearance was larger for the small tracer (3 kD) as compared to the large tracer (500 kD; P < 0.00001). WKY = 6 SHR = 6. Data are mean ± SEM.

The large difference in distribution area between SHR and WKY after passive release of tracers may influence the clearance of these tracers from the brain. Therefore, in the next set of experiments we quantified the removal of tracers from the brain. We first infused equal amounts of tracers into the hippocampus of SHR and WKY, and then allowed a 30 min period of spreading and clearance. Brain homogenates were then analysed for remaining fluorescence by spectrophotometry. Clearance was much higher for the low versus high molecular weight tracer. However, clearance of either tracer was not significantly different between SHR and WKY rats (Fig. 3).

### Pattern of tracer distribution

**Figure 4:**
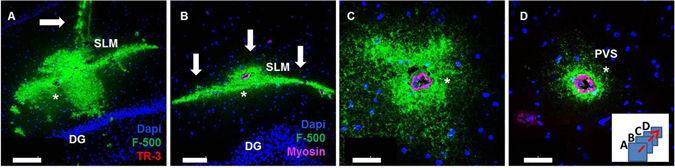
Tracer spreading within the hippocampus. Sequential sections of a SHR from the injection site (panel A; arrow indicates needle track) moving towards the caudal side of the brain (panels B–D). Tracer (500 kD) is shown in green. Panel B: 0.75 mm caudal from infusion area. Tracer spread along the SLM (arrows) and embedded arteries. Panel C: 1 mm from the infusion area, tracer co-localized with an artery. Panel D: 1.25 mm from the infusion area, stronger co-localization with the same artery. Dentate gyrus (DG); Stratum Lacunosum Molecolare (SLM); Paravascular Space (PVS). Stars indicate the same artery in the different panels. Blue: nuclear stain, magenta: smooth muscle myosin heavy chain, identifying the arterial nature of these vessels. Scale bar in A = 200 μm, B = 100 μm, C–D = 50 μm.

Tracer spreading was highly inhomogeneous. Figure 4 shows an example of tracer spreading after passive release in the hippocampus. The high molecular weight tracer spread along the stratum lacunosum moleculare (SLM) and further in the caudal direction following blood vessels, suggesting a highly preferential route for fluid movement. Based on myosin staining, these vessels were identified as arteries. Whether the tracers followed the arteries upstream or downstream could not be determined.

### Colocalization of tracers with aquaporin 4 and GFAP

**Figure 5:**
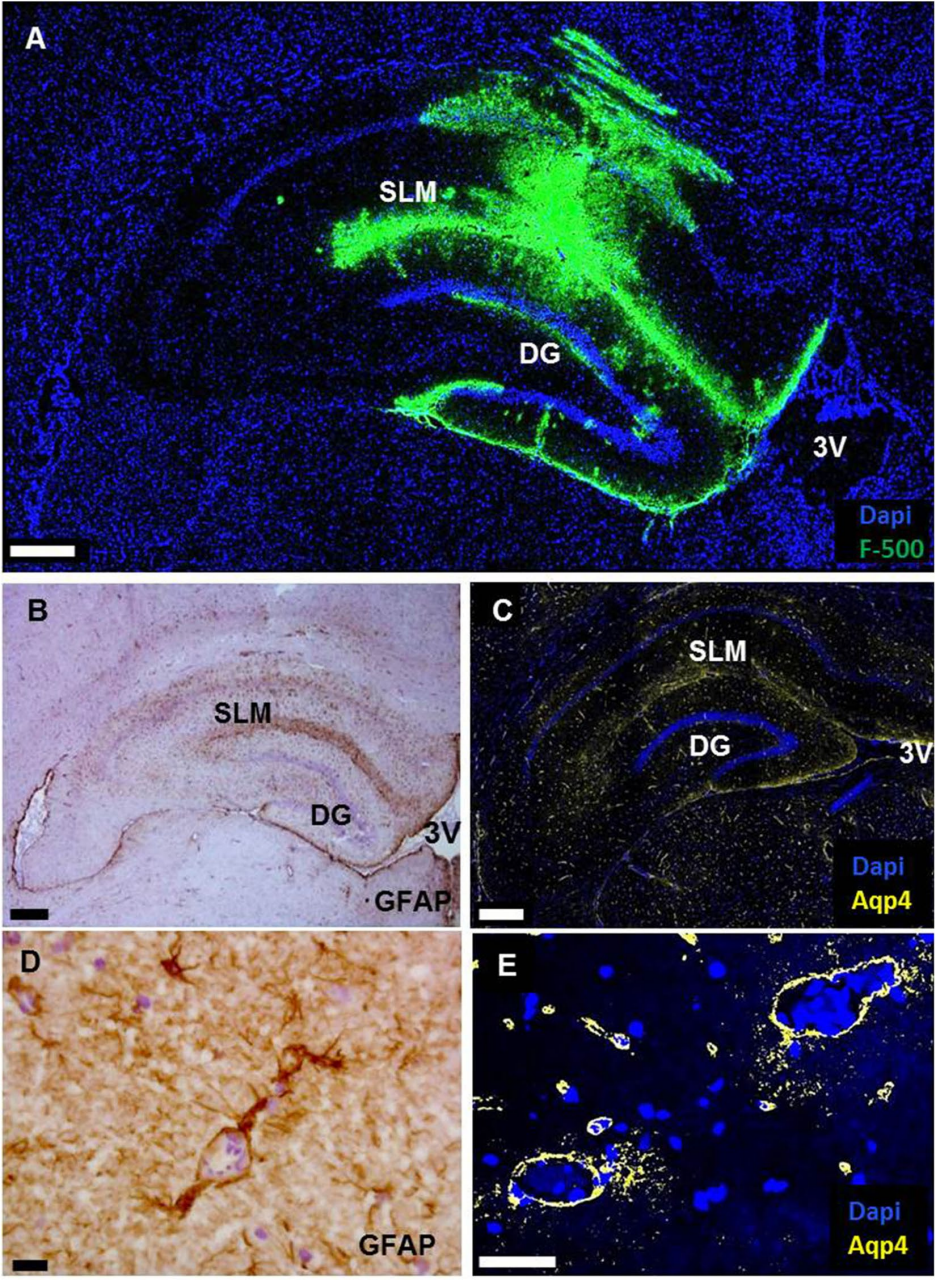
Co-localization of tracer accumulation with GFAP and aquaporin 4 staining. Panel A: High molecular weight tracer (green) distribution after infusion in the hippocampus of a WKY. Tracer accumulates along the stratum lacunosum molecolare (SLM) and at the boundaries of the hippocampus. Panel B: Expression of the astrocyte marker GFAP. Panel C: Expression of aquaporin 4. Tracer, GFAP and Aqp4 colocalize at the suprapyramidal and infrapyramidal blades of the dentate gyrus and the ependyma layer at the third ventricle. DG: dentate gyrus. 3 V: third ventricle. Scale bar in A–C 400 µm. Panels D and E show a close up of the vessels present at the SLM region. Both GFAP and Aqp4 are expressed around vessels. Scale bar in D = 15 μm, E = 50 μm.

The spreading of tracers via the SLM region and around arteries was also seen after infusion of the tracers, suggesting a low resistance pathway of ISF flow. However, in the case of infusion, tracers spread further and also accumulated at ISF-CSF borders. As these areas are known to be rich in astrocytes and their endfeet, we investigated the possible colocalization of tracer with astrocytes and aquaporin 4 (Aqp4). Indeed, we observed clear staining for the astrocyte marker GFAP and Aqp4 in areas of tracer accumulation (Fig. 5).

## Discussion

In this study we investigated the impact of hypertension on the distribution and clearance of tracers from the brains of rats. In the first set of experiments we infused tracers into the hippocampus, using a small volume and low infusion speed to reduce the impact of the infusion itself. This resulted in a non-homogeneous distribution of tracers, but no difference in the distribution area between WKY and SHR. We then followed a still more subtle approach, and allowed tracers to spread passively from the needle tip. To the best of our knowledge, this approach has not been reported before. Although the distribution area was much smaller in this case, this uncovered a large difference in distribution area in SHR as compared to WKY. We believe that the impact of infusion is an issue that has been underestimated in many studies. Infusion of 1 microliter into the brain introduces a volume of 1 mm^3^. With the extracellular space being roughly 20% of the total volume, this 1 microliter replaces the ISF of 5 mm^3^ of brain tissue (total volume of a mouse hippocampus ≈25 mm^3^, rat hippocampus ≈100 mm^3^). The endogenous production rate of ISF is unknown and difficult to determine^18^, but the results of the current study indicates that even for very limited infusion rates, endogenous transport mechanisms are easily overwhelmed.

The presence of bulk flow of ISF is debated^19^. In the current study, the low and high molecular weight tracers distributed over similar areas, which was the case in both rat strains after passive release. In SHR, there was also a strong accumulation of particularly the high molecular weight tracer in specific areas. These two features are a clear indication that solutes are dispersed via bulk flow of ISF rather than by diffusion in SHR. The larger distribution area in SHR as compared to WKY after passive release of tracers also suggests that ISF flow is markedly enhanced in the hippocampus of SHR. It should however be noted that other factors could also play a role in the difference in distribution area, such as differences in extracellular matrix composition, which could lead to sticking or retention of tracers, and differences in tortuosity of the extracellular space. A further limitation is that a direct quantitative comparison of distribution areas between the two tracers is not straightforward. First of all, the fluorophores have different optical properties. Second, the distribution area is determined not only by spreading but also by simultaneous clearance.

While the distribution area was different in size between infusion and passive release, the pattern was similar. In both cases, tracers spread and accumulated at the stratum lacunosum moleculare (SLM), around arteries, into the corpus callosum, and at the edges of the hippocampus including the border with the third ventricle. This was the case for both WKY and SHR, albeit this became only apparent after infusion in the WKY, since there was limited tracer spreading after passive release in this group. Immunostaining showed that these areas are rich in astrocytes, and strongly express aquaporin 4. Thus, it appears that ISF, containing tracers, travels from the parenchyma towards these border zones where ISF mixes with CSF. While in general the ISF-CSF border is considered less tight than the BBB, barrier properties are apparently still significant and lead to accumulation of the tracers. The presence of aquaporin 4 also suggests that facilitated water transport is necessary in these regions. This capacity may be provided by astrocytes, which are known to mediate water transport, particularly via aquaporin 4 in their endfeet^20^. It is interesting to note that arteries, but not veins, accumulated tracers. Arteries are invested with at least one, and perhaps two peri- or paravascular transport pathways^15–17, 21^. Our data indicate that in the present study, part of the tracers exited the hippocampus along arteries. This finding is consistent with data from Carare *et al*.^16^, and at variance with the view proposed by Iliff *et al*.^17^. We previously showed that these pathways around arteries connect to the CSF compartment, either at the subarachnoid space or via one of the cisterns that penetrate the brain^22^. Also in this study a similar anatomy was found, with most of the feeding arteries entering the parenchyma from the cisterns around the hippocampus. Thus, ISF appears to drain from the hippocampus into the CSF either directly via the 3^rd^ ventricle, or indirectly via cisterns and paravascular spaces around arteries.

The apparent increase in bulk flow in the SHR hippocampus raises the question what the origin of this fluid is. An obvious source for enhanced ISF flux would be leakage or secretion from the capillaries. Indeed, others have reported BBB leakage and ventricular enlargement in SHR^12, 23^. In agreement with this, we found a tendency for increased water content. This was accompanied by a shift in whole brain Na^+^/K^+^ ratio. Similar changes are seen in stroke, where an increase in brain tissue sodium concentration and a concomitant decrease in brain potassium concentration are associated with vasogenic edema^24^. Due to large concentration differences, whole brain sodium is mainly reflecting the extracellular sodium pool, whereas potassium is dominated by the intracellular pool. As we anticipate that the SHR maintains normal intra- and extracellular ion concentrations, these data point towards an expansion of the extracellular space in the brains of SHR. Although speculative, such an expansion of the extracellular space could further facilitate ISF flow due to a reduction in resistance. Taken together, the picture emerges that in SHR, BBB leakage and an increase in extracellular space are associated with enhanced flux of ISF from the capillaries into the CSF compartment.

We found that the small tracer was cleared to a larger extend than the large tracer. This was the case for both WKY and SHR. Tracers may have been eliminated across the BBB, via the ISF into the CSF, and possibly via the choroid plexus as noted previously^15, 25^. However, in view of the enhanced spreading of tracers in SHR, the absence of a difference in clearance between WKY and SHR for both tracers appears to be a contradictory finding. One possibility is that infusion of a relatively large volume of tracers overwhelms endogenous clearance mechanisms, which obscures subtle differences in clearance rate between SHR and WKY. This would be a situation similar to the tracer spreading results in this study. Alternatively, enhanced ISF flow may not necessarily lead to enhanced solute removal. In that case, we speculate that enhanced ISF flow relates to an increase in water and ion fluxes, but not necessarily leads to more washout of larger molecules. These molecules may be retained within the tissue at barrier sites. Indeed, we observed strong accumulation of the tracers, particularly of the high molecular weight tracer, at the borders of ISF and CSF exchange. We therefore interpret these findings as a sieving effect, resulting from relatively easy passage of water and ions, and accumulation of tracer. Whether such a mechanism is relevant for accumulation of endogenous waste products remains to be established. It is however very tempting to speculate on such a phenomenon, as tracer accumulation as observed in our experiments mirrors the pattern of amyloid β accumulation in the hippocampus of a mouse model of Alzheimer’s disease^26^.

Studies on human brain interstitial fluid flux and hypertension are scarce. Enlarged perivascular spaces are considered a marker for impaired ISF drainage^27^ and correlate with vascular amyloid β deposition^28^. White matter hyperintensities and enlarged perivascular spaces are also associated with dementia and may be explained by impaired drainage of ISF^29^. Another study using MRI suggested that systolic hypertension is associated with an increase in extracellular fluid^30^. Thus, there is only circumstantial evidence that ISF flow is altered in dementia and potentially aggravated by hypertension. Currently, treatment options therefore seem premature and await future studies that shed more light on ISF production, pathways, and impact of hypertension on clearance of waste products from the brain.

To conclude, we showed that ISF flow is greatly enhanced in the hippocampus of SHR rats as compared to normotensive WKY. In these animals, tracer distribution is consistent with bulk flow, rather than diffusion. This is based on the notion that high and low molecular weight tracers spread similarly, and strongly accumulate at border zones of ISF-CSF exchange. The retention of tracers in these areas can be explained by a sieving effect on ISF, which could be relevant for the accumulation of endogenous solutes and waste products such as amyloid β.

## Materials and Methods

### Animals

For this study 39 animals were used. Male normotensive Wistar Kyoto rats (WKY/NCrl) (n = 20) and male spontaneously hypertensive rats (SHR/NCrl) (n = 19) were obtained from Charles River at the age of 11 weeks. Animals were kept until 8 months of age, housed in groups and fed *ad libitum* with standard laboratory food and free access to water. Experiments were done during daytime, which is the sleep phase for rats. One animal died during anesthesia, before the start of the experiment. All experimental protocols were approved by the Committee for Animal Experiments of the Academic Medical Center Amsterdam, and in accordance with the European Communities’ Council Directive 2010/63/EU.

### Blood pressure measurements

Blood pressure (BP) was measured non-invasively with a tail-cuff system (Kent Scientific, Torrington, Connecticut, USA). Rats were accustomed to handling and to the restrainer during a training period of 4 days before the BP measurements. During the measurements, the animals were placed in the restrainer on a heating pad, for at least 10 minutes to warm up the tail. In each animal, 4 to 10 BP measurements were made and averaged. In a subset of animals we also measured BP under KDA (see below) anaesthesia. This revealed no difference in BP between anaesthetized and awake animals (data not shown).

### Anaesthesia

In initial experiments, where tracers were infused into the brain by pumping, rats were anaesthetized by intraperitoneal injection of 2 ml/kg of KDA mix, consisting of a combination of ketamine (75 mg/kg, Nimatek, Eurovet), dexdomitor (0.5 mg/kg, Orion Pharma), and atropine (0.05 mg/kg, atropine sulphate, Eurovet) dissolved in PBS (Phosphate Buffered Saline, Lonza). Additional oxygen (99%) was administered via a nose cap to prevent hypoxia. As we experienced a more stable and adjustable level of anaesthesia with isoflurane, the remainder of the study was carried out with animals under anaesthesia with isoflurane (3% in O_2_) applied via an inhalation mask.

### General surgical procedure

The animals were anaesthetized and the scalp was shaved. Subsequently, heads were immobilized in a stereotactic frame (Stoelting) and the core body temperature was maintained using a heating pad. Ocular lubricant ointment (Duratears®, Alcon) was applied to keep the eyes hydrated. The temperature was monitored with a rectal thermometer (Greisinger Electronics) during the procedure. A small longitudinal skin incision was made on the skull and 10% xylocaine (AstraZeneca B.V.) was provided as additional local anaesthesia. The periosteum was scratched off using a scalpel. Subsequently, according to the Paxinos and Watson Rat Brain atlas (6^th^ edition, 2007), we defined the stereotactic coordinates for the CA1 region of the hippocampus as −4.0 mm caudal, 2.0 mm lateral, and 4.0 mm deep, from the bregma point. The depth was calculated taking into account the thickness of the skull. A small burr hole was drilled with a dental drill (W&H). Subsequently, a 33-gauge needle (Hamilton) connected to a polyethylene catheter was inserted into the hippocampus using the stereotactic device. The catheter was connected to a syringe filled with a mixture of green (500 kDa) and red (3 kDa) fluorescent dextran. Both tracers were used at a final concentration of 10 mg/mL.

### Infusion of tracers into the hippocampus

In this set of experiments, rats were anaesthetized with KDA mix. We infused 2 μl of the dextran mixture at a controlled flow rate of 0.066 μl/min using a syringe pump (Harvard Apparatus, Holliston, MA, USA), over a 30 minute period. After infusion, the syringe pump was stopped and the needle was removed 1 minute after the end of the infusion period. Subsequently, the animals were euthanized with an overdose of the anaesthetic and rapidly decapitated, after which the brains were carefully dissected. The brains were weighed and cut into three coronal blocks using an adult rat brain slicer matrix (Zinc instruments). The blocks were separately embedded in Tissue-Tek® (Sakura), snap frozen in liquid nitrogen, and stored at −80 °C. The brains were then cut in coronal sections, 5 µm thick, using a cryostat (Microm HM 560) and collected on SuperFrost slides (Menzel-Gläzer). After sectioning, sections were stored at −80 °C until they were used for immunohistochemistry.

### Passive tracer release in the hippocampus

In the remainder of the study, animals were anesthetized with isoflurane. For this set of experiments we inserted the needle via the burr hole and we let the tracers diffuse from the tip of the needle into the rat hippocampus for 30 minutes. At the end of the diffusion period the animals were euthanized and the brain processed as described for the infusion set of experiments.

### Confocal imaging

Prior to imaging brain slices were fixated in 3.7% paraformaldehyde (PFA) for 30 minutes. Then, cell nuclei were stained with bisbenzimide (Sigma). Selected slices were additionally stained to identify specific cell types and proteins. To discriminate between arteries and veins we stained for myosin heavy chain. Antibodies against GFAP and aquaporin 4 (Aqp4) were used to stain astrocytes and water channels. Fluorescent images were acquired using a confocal laser-scanning microscope (Leica TCS SP8), with a 20x objective for details and 10x objective for overviews. ImageJ software was used to quantify the distribution area of the tracers. Care was taken to prevent pixel saturation and to apply the same confocal settings to sections within each group of experiments.

### Clearance of tracer from the hippocampus

To quantify the clearance we first infused a known volume (2 μl) of the dextran mixture at 0.066 μl/min for 30 min. Then, we allowed a subsequent 30 min period for spreading and clearance of the tracers. We used active infusion of tracer in this set of experiments, because measuring clearance requires a similar and known quantity of tracer to start with in WKY and SHR. Animals were then euthanized rapidly and brains were carefully dissected and weighed. Brains were subsequently homogenized in 7 ml of RIPA buffer, using a manual potter and an automated blender to obtain a homogenous suspension. Fluorescence spectrometry was then performed and the total amount of both dyes in the brain homogenate was determined from integration of the spectra over the appropriate wavelengths. In order to normalize for possible differences in turbidity in the different samples, the spectrometry was repeated after addition of a known quantity of the tracers to the samples.

### Brain ashing procedure

We used snap frozen brain samples to analyze water, potassium, and sodium content. To determine brain water content, we compared brain sample weights before and after desiccation at 90 °C for 48 hours. Next, samples were dry ashed for 40 hours at 450 °C. After ashing, all samples were dissolved in 5% HNO3. Sodium and potassium concentrations were determined by flame photometry.

### Reagents

Dextran, Texas Red-labelled (3 kD, Ex. 595 nm/Em. 615 nm) and dextran, fluorescein-labelled (500 kD, Ex. 494 nm/Em. 521), both lysine-fixable, were purchased from Molecular Probes-Life Technologies (Eugene, OR, USA). These dyes were dissolved in artificial cerebrospinal fluid (aCSF). In the passive release experiments we increased the final concentration of FITC dextran to 50 mg/ml. For immunohistochemistry we used antibodies against: smooth muscle myosin heavy chain 11 antibody (Abcam), Aqp4 (Millipore) and GFAP (Dako Cytomation, Glostrup). Cell nuclei were stained with bisbenzimide (Sigma). Slices were mounted in fluorescent mounting medium (Dako). RIPA buffer consisted of 150 mM sodium chloride, 1.0% Triton X-100, 0.5% sodium deoxycholate, 0.1% SDS, 1 mM EDTA, in 50 mM Tris, pH 8.0.

### Statistics

Data are expressed as mean ± SEM. Data were analysed using Students’ t-test or two-way ANOVA. A *p* value of <0.05 was considered statistically significant.
